# Ether and Chloroform; Their Employment in Surgery, Dentistry, Midwifery, Therapeutics, &c.

**Published:** 1851-04

**Authors:** 


*Ether and Chloroform; their Employment in Surgery, Dentistry, Midwifery, Therapeutics, &c.* By J. F. B. Flagg, M D., Surgeon Dentist, Member of the Rhode Island Medical Society. Philadelphia: Lindsay & Blakiston, 12mo., pp. 189, 1851.

The above beautifully gotten up work contains a great deal of interesting information with regard to the anæsthetic effects of ether and chloroform; and to those who are desirous of administering agents of this sort, it may be consulted with advantage. In the practice of surgery and obstetrics they have been extensively used, and, very generally, with the most happy and gratifying results; but in dentistry they have been less frequently employed.

